# Automating the Identification of Feedback Quality Criteria and the CanMEDS Roles in Written Feedback Comments Using Natural Language Processing

**DOI:** 10.5334/pme.1056

**Published:** 2023-12-18

**Authors:** Sofie Van Ostaeyen, Loic De Langhe, Orphée De Clercq, Mieke Embo, Tammy Schellens, Martin Valcke

**Affiliations:** 1 Department of Educational Sciences at Ghent University, Belgium; 2 Language and Translation Technology Team at Ghent University, Belgium; 3 Department of Educational Sciences at Ghent University and in the Expertise Network Health and Care at the Artevelde University of Applied Sciences, Belgium

## Abstract

**Introduction:**

Manually analysing the quality of large amounts of written feedback comments is time-consuming and demands extensive resources and human effort. Therefore, this study aimed to explore whether a state-of-the-art large language model (LLM) could be fine-tuned to identify the presence of four literature-derived feedback quality criteria (*performance, judgment, elaboration* and *improvement*) and the seven CanMEDS roles (*Medical Expert, Communicator, Collaborator, Leader, Health Advocate, Scholar* and *Professional*) in written feedback comments.

**Methods:**

A set of 2,349 labelled feedback comments of five healthcare educational programs in Flanders (Belgium) (specialistic medicine, general practice, midwifery, speech therapy and occupational therapy) was split into 12,452 sentences to create two datasets for the machine learning analysis. The Dutch BERT models BERTje and RobBERT were used to train four multiclass-multilabel classification models: two to identify the four feedback quality criteria and two to identify the seven CanMEDS roles.

**Results:**

The classification models trained with BERTje and RobBERT to predict the presence of the four feedback quality criteria attained macro average F1-scores of 0.73 and 0.76, respectively. The F1-score of the model predicting the presence of the CanMEDS roles trained with BERTje was 0.71 and 0.72 with RobBERT.

**Discussion:**

The results showed that a state-of-the-art LLM is able to identify the presence of the four feedback quality criteria and the CanMEDS roles in written feedback comments. This implies that the quality analysis of written feedback comments can be automated using an LLM, leading to savings of time and resources.

## Introduction

In healthcare education, the ongoing move to competency-based education (CBE), milestones, entrustable professional activities (EPAs) and mastery learning has challenged assessment approaches [1]. Integral to CBE is that healthcare professionals observe students’ performance on authentic tasks and provide specific feedback across a predetermined competency framework [2,3,4]. This implies a higher demand for descriptive, narrative and actionable feedback [5,6]. Consequently, the traditional focus on quantitative scoring no longer fits the needs of contemporary assessment conceptions [6,7]. Therefore, increased attention is paid to the potential of written feedback comments regarding student’s performance during clinical placements [8].

High-quality written feedback comments are acknowledged as rich and valid data sources to direct and support self-regulated learning, remediation and decision-making during clinical placements [6,9,10]. Feedback comments are considered of high quality when they meet the following four quality criteria: (1) they describe the student’s *performance* on which the feedback is provided [11], (2) include a *judgment* to denote the gap between this performance and a standard [11], (3) contain an *elaboration* statement that builds further on the judgment [12] and (4) provide strategies on how the student’s performance can be *improved* [8]. Furthermore, in view of CBE, high-quality feedback needs to provide a comprehensive overview of the student’s competency development to allow for valid decision-making [7,13]. This implies that feedback comments need to be aligned with the roles and underlying competencies that graduating healthcare professionals should adopt and develop [7]. These competencies are defined following competency frameworks such as the Canadian Medical Education Directions for Specialists (CanMEDS) framework, which outlines seven roles that together represent a holistically competent physician: *Medical Expert, Communicator, Collaborator, Leader, Health Advocate, Scholar and Professional* [14]. The latter framework was originally developed to define the competence of physicians, but has also been validated in the context of other healthcare professions [15].

The literature extensively reports on the criteria for high-quality feedback [16,17]. Nevertheless, it remains difficult for feedback providers to apply these quality criteria in practice, resulting in an overall low quality of written feedback comments [18,19]. Healthcare students report feedback comments as being nonspecific and too generic [20]. Previous research reveals a lack of feedback skills among feedback providers [21], as they have difficulty using feedback forms accurately, face challenges in using defined learning outcomes as criteria for assessing students’ competencies, and struggle to provide high-quality feedback even after training [21,22,23].

Given the lack of high-quality written feedback comments in healthcare education, feedback providers might benefit from receiving timely and constructive feedback on their feedback comments. This could enable them to enhance their feedback skills and provide more high-quality feedback comments in the future [24]. However, human evaluation of the quality of a large amount of feedback comments is time-consuming and demands extensive resources and human effort [25]. This is a growing problem as the expansion of digital tools to facilitate feedback delivery has caused an intensification in the quantity of written feedback comments. For example, ePortfolios are frequently used during clinical placements to support students in seeking feedback and teachers and clinical mentors in giving feedback [26,27].

One way to overcome this challenge could be to leverage technological advances in the field of artificial intelligence (AI), which is a branch of computer science dealing with the replication of intelligent behaviour by computers [28]. A subfield of AI, known as Natural Language Processing (NLP), uses machine learning techniques* (See Appendix A for a further description of terms marked with *) to make it possible for computers to understand and process human language as humans do [29]. In healthcare, NLP techniques have been demonstrated to provide near real-time data analysis of large complex qualitative datasets [30]. Comparably, NLP might be helpful to evaluate large amounts of written feedback comments in a short amount of time [5].

Few studies in the field of healthcare education explored NLP techniques to classify feedback quality [25,31,32]. The focus of available studies mainly targeted the classification of feedback quality using traditional NLP techniques and algorithms* (e.g., random forest, naïve bayes, gradient boosted trees, logistic regressions and support vector machines). The latter rely on hand-crafted features* for model* training, which often require significant human labelling* and conceptualisation effort. However, more recently, paradigms in the field of NLP steadily shifted towards the creation of contextual language representations* using deep neural networks*, requiring a minimum of human intervention in the classification process [33]. These contextual language representations result in large language models (LLMs), which can be fine-tuned* on a limited amount of human-annotated training data to perform a specific task. A contemporary application that uses such an LLM is the chatbot ChatGPT, with GPT3.5 as its backbone [34]. To our knowledge, this paper presents the first study to explore whether a state-of-the-art LLM can be fine-tuned to identify the presence of the four feedback quality criteria and the seven CanMEDS roles in written feedback comments. To this end, we pose two research questions:

1. To what extent can a state-of-the-art LLM identify the presence of feedback quality criteria in written feedback comments?
2. To what extent can a state-of-the-art LLM identify the presence of the CanMEDS roles in written feedback comments?

## Methods

### Data collection and labelling

The research data consisted of 2,349 written feedback comments retrieved from healthcare students’ ePortfolios. These feedback comments were collected in June 2021 in the context of five healthcare educational programs: specialistic medicine (postgraduate), general practice (postgraduate), midwifery (undergraduate), speech therapy (undergraduate) and occupational therapy (undergraduate). Feedback comments were qualitative free texts entered into an ePortfolio (Medbook) by a teacher or clinical mentor who guided the student during the clinical placement. In a prior study [35], three researchers (SVO, SJA and OJ) manually labelled the written feedback comments to investigate their quality, and how these feedback comments were aligned with the CanMEDS roles. By screening these feedback comments, the researchers identified the presence of predefined feedback quality criteria (*performance, judgment, elaboration* and *improvement*) (Appendix B) and the CanMEDS roles (*Medical Expert, Communicator, Collaborator, Leader, Health Advocate, Scholar, Professional*) [36]. With an average Cohen’s Kappa value of 0.63 for the feedback quality criteria and one of 0.53 for the CanMEDS roles, moderate to substantial agreement was achieved [37] (see Appendix C for the exact Cohen’s Kappa values per label). For the present study, these labelled feedback comments served as data to fine-tune the LLMs, which will be explained in the next sections.

Ethical approval was obtained from the Ethical Committee of the Faculty of Psychology and Educational Sciences of Ghent University (reference #2021-34) and a Data Transfer Agreement was signed to legally document the data exchange between the Medbook company and Ghent University. The researchers used the CanMEDS framework after obtaining permission from the Royal College of Physicians and Surgeons of Canada.

### Dataset overview

The 2,349 labelled feedback comments were subsequently split into 12,452 sentences. With these sentences, two datasets were created to answer the two research questions. The first dataset indicated for each sentence whether the four feedback quality criteria were present or not. To this purpose, the integers ‘1’ (present) and ‘0’ (absent) were used. Similarly, the second dataset indicated whether the seven CanMEDS roles were present in the sentences or not, using the same binary labels. In view of the machine learning analysis, each dataset was randomly split into a training, development and test dataset, including respectively 70% (n = 8,716), 15% (n = 1,868) and 15% (n = 1,868) of the sentences. This data partition strategy in a training, development and test dataset is the most common approach when performing machine learning analyses [29]. The training dataset is used to deduce the knowledge required to train and fine-tune the LLM to a specific task. The development dataset is used to optimise the parameters of the model. The test dataset is used as unseen data to evaluate the model.

The table in Appendix D summarises the distribution of the labelled feedback quality criteria and CanMEDs roles in the training, development and test datasets. As can be derived from this table, the datasets were skewed with some labels occurring to a larger extent than others. Regarding the feedback quality criteria, the *elaboration* criterion was present in considerably fewer sentences compared to the other criteria. In terms of the CanMEDS roles, the *Medical Expert* role appeared in many sentences while other roles such as *Leader, Health Advocate* and *Professional* were present in a smaller proportion of the sentences.

### Machine learning analysis

**Figure 1:**
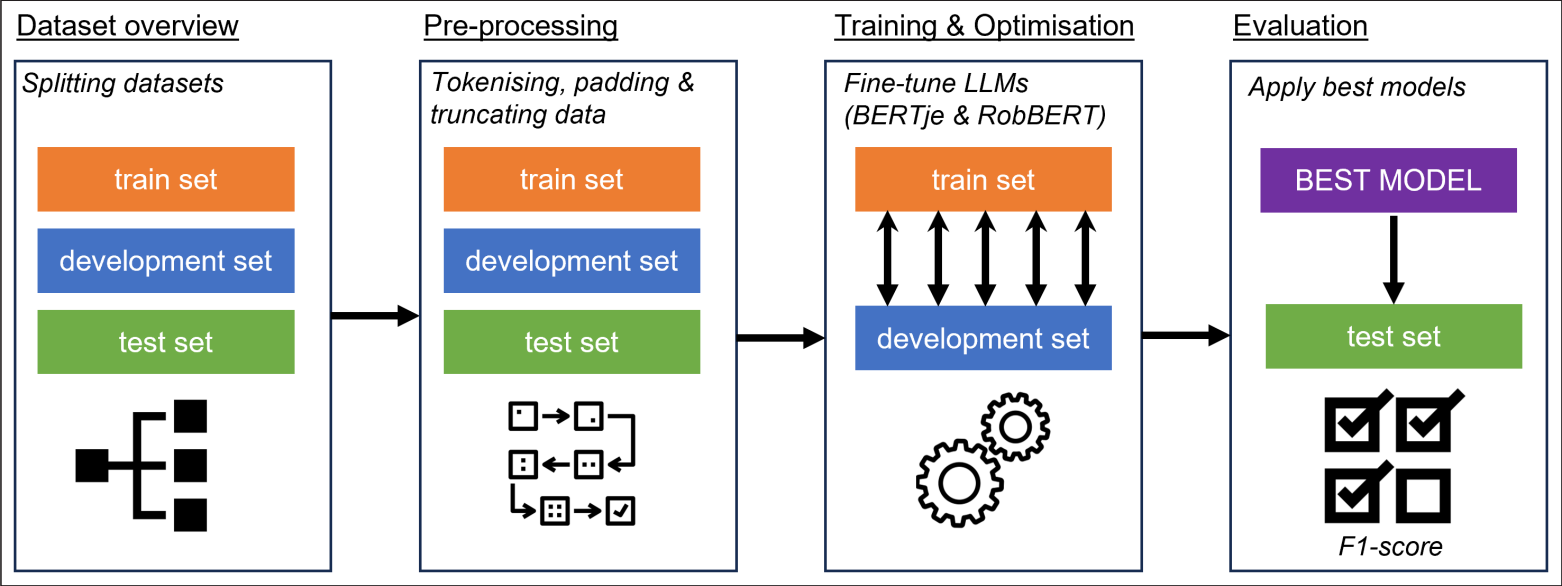
Flowchart depicting the workflow of the research method.

The machine learning analysis was conducted in October 2022. Figure 1 depicts the steps followed throughout the analysis phase. In this study, one sentence could receive multiple labels (e.g. multiple feedback quality criteria or multiple CanMEDS roles). This implied a model being able to make multiple predictions* for one sentence. Multiclass-multilabel classification models are appropriate for this purpose. These models can make a prediction for each sentence in the form of a binary decision, indicating the sentence gets a particular label or not. Four multiclass-multilabel classification models were trained: two to identify the four feedback quality criteria and two to identify the seven CanMEDS roles.

### Model selection

This study focused on the use of an LLM to train the four classification models. In view of this, a transformer language model was selected. Transformer language models are neural networks that are currently becoming the most common architecture to develop and train language models. They quickly replaced earlier machine learning architectures for completing various NLP tasks [38].

The state-of-the-art transformer language model BERT (Bidirectional Encoder Representations From Transformers) [39] has been revolutionary for NLP research and applications. It has been proven that transformer models with an encoder architecture*, such as BERT, are particularly suitable for sentence classification [40]. These neural architectures are first initialised using large-scale unlabelled corpora (pre-training) and subsequently trained on labelled data for a specific task (fine-tuning). To pre-train BERT, Devlin and colleagues [39] used the BookCorpus (800 M words) and English Wikipedia (2.500 M words). Since BERT was trained on English data, it is hardly directly applicable to other languages. A multilingual BERT model exists, but language-specific models are expected to result in superior performance [41]. Therefore, monolingual models with the BERT architecture were developed for different languages (e.g., Italian: AlBERTo [42]).

As our research data were written in Dutch, we used two Dutch BERT models: BERTje [41] and RobBERT [43], released in 2019 and 2020 respectively. The primary distinction between these two models is the size of the corpus utilised for pre-training. The corpus used to train BERTje was about 12 GB, compared to a corpus size of 39 GB to train RobBERT [43]. Additionally, the BERTje model was pre-trained with both Masked Language Modelling* (MLM) and Next Sentence Prediction* (NSP) tasks, while pre-training for RobBERT remained restricted to the MLM task.

### Model training and optimisation

To train the four classification models (two with BERTje and two with RobBERT), we undertook the following steps for each model. The first step was to pre-process the data. In the present study, this involved tokenising the data. Tokenisation includes breaking down the data into tokens* ensuring the data is converted into a format that can be processed by the transformer language models. Additionally, the neural transformer architecture requires that input sentences have the same pre-defined number of tokens (n = 512). In order to enforce this, sentences were padded and truncated where needed, without affecting the language context. With padding, additional tokens were added to the sentences until they reached the required token length. Truncating works in the other direction by truncating long sentences. The second step was to define the model’s configuration for fine-tuning. This involved selecting different hyperparameters* that were improved in different optimisation runs and evaluating the results on the development dataset. Five optimisation runs were done after which the hyperparameters of the best run were selected for the model. In the third step, the final classification model was trained with the best hyperparameters. The fourth step involved using the final model to predict labels in the test dataset.

### Model evaluation

To evaluate the performance of classification models, different metrics can be used. We first considered accuracy. This metric measures the number of correct predictions as a percentage of the predictions that are made. However, the accuracy metric is only useful when the labels in the dataset are equally distributed. As previously mentioned, the dataset used in this study was skewed (see Appendix D) which is why it is preferable to use the F1-score as the evaluation metric. F1-scores take into account the type of error made, in addition to the number of errors made by the model [44]. The F1-score reflects the harmonic mean of precision and recall (see formula below) and ranges from 0 to 1, where 0 indicates poor performance and 1 indicates perfect performance on the classification task. Precision and recall are the two most common metrics accounting for class imbalance. Precision refers to the proportion of true positive predictions (correctly identified examples belonging to a label) among all predicted positive examples (including also falsely identified examples). So, precision measures how accurate the model is when predicting the presence of a label. Recall refers to the proportion of true positive predictions among all actual positive examples in the dataset. In this way, recall measures the model’s ability to identify all positive examples in the dataset. In the F1-score the average of precision and recall is calculated by using the following formula:


\[F1score = 2*\frac{{Precision*Recall}}{{Precision + Recall}}\]

These three metrics were thus calculated for each label in isolation. However, to also get a general overview of the overall performance these F1-scores per class were also macro-averaged, implying that all classes were treated equally and independently of one another.

## Results

### Identifying the presence of feedback quality criteria in written feedback comments

The classification models predicting the presence of the four feedback quality criteria trained with BERTje and RobBERT achieved macro average F1-scores of 0.73 and 0.76 respectively. Table 1 summarises the evaluation metrics for each of the quality criteria individually.

**Table 1:** Performance of the models trained with BERTje and RobBERT predicting the presence of the quality criteria in the feedback comments.


	BERTje			ROBBERT		
	
	PRECISION	RECALL	F1-SCORE	PRECISION	RECALL	F1-SCORE

*Performance*	0.86	0.88	0.87	0.86	0.89	0.87

*Judgement*	0.83	0.86	0.85	0.85	0.89	0.87

*Elaboration*	0.40	0.30	0.35	0.40	0.43	0.41

*Improvement*	0.86	0.88	0.87	0.85	0.89	0.87


As can be observed, the F1-scores for the individual quality criteria *performance, judgment* and *improvement* showed values equal to or above 0.85, indicating a comparatively good performance on the classification task for each model. The performance of the models for the prediction of the *elaboration* criterion was rather low, as the value for the F1-score of the model trained with BERTje was 0.35 and with RobBERT 0.41.

### Identifying the presence of the CanMEDS roles in written feedback comments

The macro average F1-score of the classification models predicting the presence of the CanMEDS roles achieved a value of 0.71 for BERTje and of 0.72 for RobBERT. Table 2 provides the evaluation metrics for each of the CanMEDS roles and reveals that the F1-scores for these individual roles varied between 0.58 and 0.85. The F1-scores of the role *Medical Expert* were the highest (0.84 for BERTje and 0.85 for RobBERT), followed by those of the roles *Leader* (0.74 for BERTje and 0.79 for RobBERT), *Health Advocate* (0.75 for BERTje and 0.73 for RobBERT), *Scholar* (0.71 for BERTje and 0.72 for RobBERT) and *Collaborator* (0.67 for BERTje and 0.71 for RobBERT). For the roles *Communicator* and *Professional*, the F1-scores were respectively 0.66 and 0.61 for BERTje and 0.66 and 0.58 for RobBERT.

**Table 2:** Performance of the models trained with BERTje and RobBERT predicting the presence of the CanMEDS roles in the feedback comments.


	BERTje			ROBBERT		
	
	PRECISION	RECALL	F1-SCORE	PRECISION	RECALL	F1-SCORE

*Medical Expert*	0.83	0.86	0.84	0.84	0.85	0.85

*Communicator*	0.70	0.63	0.66	0.70	0.63	0.66

*Collaborator*	0.69	0.66	0.67	0.72	0.71	0.71

*Scholar*	0.77	0.65	0.71	0.74	0.71	0.72

*Leader*	0.80	0.68	0.74	0.82	0.77	0.79

*Health Advocate*	0.81	0.70	0.75	0.81	0.67	0.73

*Professional*	0.69	0.55	0.61	0.67	0.51	0.58


## Discussion

The present study aimed to explore to what extent a state-of-the-art LLM could identify the presence of the four feedback quality criteria and the seven CanMEDS roles in written feedback comments. Therefore, a set of 2,349 feedback comments of five healthcare educational programs was labelled and split into 12,452 sentences. The Dutch BERT models BERTje and RobBERT were used to train four multiclass-multilabel classification models. The classification models predicting the presence of the four quality criteria trained with BERTje and RobBERT achieved a macro average F1-score of 0.73 and 0.76 respectively. The F1-score of the model predicting the presence of the CanMEDS roles trained with BERTje showed a value of 0.71 and with RobBERT a value of 0.72. This means that a state-of-the-art LLM, such as a transformer language model, is relatively apt to identify the feedback quality criteria and the CanMEDS roles in written feedback comments. In this case, the RobBERT model performed slightly better in both identifying the quality criteria and the CanMEDS roles, which is in line with previous studies on other sentence classification tasks [43].

The number of studies that focus on using NLP techniques to classify feedback quality in the context of healthcare education is limited [25,31,32]. Furthermore, these studies only used feedback comments from one educational program (anesthesiology [25] and surgery [31,32]) to train NLP models. In contrast, this study contributes to the existing knowledge about AI-driven feedback quality classification by using a dataset consisting of feedback comments from different educational programs in healthcare. The results of this study indicate that LLMs can be used in the context of other healthcare educational programs to classify feedback quality.

The prediction of the individual feedback quality criteria achieved high F1-scores, except for the *elaboration* criterion (0.35 for BERTje and 0.41 for RobBERT). The suboptimal F1-scores for the prediction of this criterion were a result of the lower values for precision and recall (respectively 0.40 and 0.30 for BERTje and 0.40 and 0.43 for RobBERT), which means the models did misclassify a number of sentences and missed a number of correct classifications. In previous work [35] this ambiguity in the prediction of the *elaboration* criterion also emerged in the manual analysis (Cohen’s Kappa value of 0.24 in Appendix C). Furthermore, the manual labelling of the feedback comments showed that the *elaboration* criterion was only present in a limited number of feedback sentences (n = 897; 7.20%). Consequently, fewer training data were available for the classification models to deduce knowledge from (n = 622; 7.14%), which might have impacted the ability of the models to accurately classify unseen examples.

Similarly, the F1-scores of the classification of the CanMEDS roles achieved high values. However, the F1-scores of the roles *Communicator* and *Professional* were rather low (respectively 0.66 and 0.61 for BERTje and 0.66 and 0.58 for RobBERT). Notwithstanding the fact that many sentences did contain information related to the *Communicator* role, F1-scores were lower compared to F1-scores related to other roles. These lower F1-scores can be explained by the fact that the models were unable to identify the *Communicator* role in some sentences due to the implicit nature of the information, rather than being literal. Furthermore, the models identified the *Communicator* role in sentences containing words related to communication (e.g., conversation, contact, saying, speaking) while these words referred to another role. Specifically, the models struggled to distinguish the *Communicator* role from the *Collaborator* role. The CanMEDS framework differentiates between these two roles: the *Communicator* role refers to the healthcare professional’s communication with patients and families and the *Collaborator* role refers to the collaboration and communication with colleagues and other healthcare professionals [14]. As the sentences not always explicitly stated whether the communication was with a patient or a colleague, the models have misclassified these sentences. In addition, some aspects of the *Health Advocate* role are closely aligned with the *Communicator* role, as the first key competency of the *Health Advocate* role focuses on the ability to respond to the needs of individual patients by incorporating disease prevention, health promotion and health surveillance into interactions with individual patients [14].

Identifying the *Professional* role in feedback comments appears to be difficult for both humans (Cohen’s Kappa value of 0.21 in Appendix C) and computers. A first explanation for the lower performance of the classification models for the *Professional* role may be the availability of fewer training data for this role. This might explain why the models struggled to accurately categorise sentences reflecting the *Professional* role. However, this is in contrast to the findings in relation to the roles *Leader* and *Health Advocate*. For these roles, fewer sentences were available but the F1-scores for these roles achieved higher values. A second explanation for the low F1-scores of the *Professional* role is that the underlying key and enabling competencies of the CanMEDS roles overlap in practice and complement each other [45]. Particularly the role *Professional* overlaps with the roles *Communicator, Collaborator, Leader* and *Health Advocate* [46].

The results of this study are encouraging as they indicate the potential of NLP techniques to assist in analysing large amounts of written feedback comments. This provides opportunities for healthcare education to utilise LLMs in practical applications that facilitate feedback provision. Therefore, in subsequent work, the two classification models trained with RobBERT will be incorporated into an intelligent just-in-time feedback support tool that can be seamlessly integrated into a digital feedback platform, such as an ePortfolio. The feedback tool enables feedback providers to evaluate the quality and alignment with the CanMEDS roles of their feedback comments at the click of a button during the writing process. The outcomes of this AI-assisted evaluation will be presented to the feedback provider as a detailed report within the feedback platform. This report highlights any missing quality criteria and CanMEDS roles in the feedback comment. Additionally, the tool will offer adaptive tips that pertain to the missing quality criteria and CanMEDS roles, allowing feedback providers to modify the feedback comment before saving it in the feedback platform. In this way, students do not have access to the feedback provider’s modifications, but only to the final version of the feedback comment. In future research, we will investigate the practical implementation of such an AI-based feedback tool in the clinical learning environment and its impact on the quality and alignment with the CanMEDS roles of written feedback comments.

The present study reflects some limitations. First, the researchers did not collect demographic data about feedback providers. Previous research found that feedback quality could be associated with the gender of the feedback provider [47]. This may have caused bias in feedback comments and subsequently in the NLP model output. Second, although the research data contained feedback comments from different healthcare educational programs, the results cannot be generalised to other educational programs within healthcare or beyond. However, previous research has demonstrated the potential for applying NLP techniques on feedback comments from other educational programs [48]. A third limitation is that a classification model cannot deal with nonliteral language. Previous research pointed out feedback providers use a hidden code and hedging strategies in their assessment language [49,50]. Future research should investigate how NLP techniques could deal with such textual complexity. A fourth limitation is related to the quality of the labelled data. For the *elaboration* criterion and the CanMEDS role *Professional* the Cohen’s Kappa values were rather low (respectively 0.24 and 0.21), although they did still indicate fair agreement. While discrepancies were discussed and resolved, it is possible that noise was introduced into the data which led to misclassification of the models.

To advance the use of NLP techniques in healthcare education, this study explored the utility of an advanced machine learning architecture to identify the presence of four feedback quality criteria and the seven CanMEDS roles in written feedback comments. The results show that LLMs can be fine-tuned to perform such classification tasks. This indicates that with the use of an LLM, the quality analysis of written feedback comments can be automated, leading to savings in terms of both time and resources. Future research should focus on finding ways to incorporate the evidence on the effectiveness of NLP techniques for analysing written feedback comments into usable applications.

## Data Accessibility Statement

The participants of this study did not give written consent for their data to be shared publicly, so due to the sensitive nature of the research supporting data is not available.

## Additional Files

The additional files for this article can be found as follows:

10.5334/pme.1056.s1 Appendix A. Glossary.

10.5334/pme.1056.s2 Appendix B. Structured categorisation matrix quality criteria.

10.5334/pme.1056.s3 Appendix C. Cohen’s Kappa values for manual labelling.

10.5334/pme.1056.s4 Appendix D. Data distribution regarding the feedback quality criteria and CanMEDS roles throughout the training, development and test dataset.
