# Supplementary material for: Automating the Identification of Feedback Quality Criteria and the CanMEDS Roles in Written Feedback Comments Using Natural Language Processing
Source: Perspect Med Educ. 2023 Dec 18;12(1):540–9. doi: 10.5334/pme.1056 (PMC10742245; doi:10.5334/pme.1056)
Supplement: Appendix A. — Glossary. [file pme-12-1-1056-s1.pdf]

Appendix A. Glossary

**Word**	**Explanation**
Algorithm	An algorithm is a set of instructions or procedures used to solve a specific problem or perform a particular task. Algorithms are used extensively in machine learning. Machine learning algorithms can be supervised, unsupervised, or semi-supervised, depending on whether or not they are provided with labelled data to guide their learning.
Contextual language representations	Contextual language representations are numerical representations of words, phrases, or sentences that capture the context in which they appear. These representations are generated by deep learning models, such as transformer models, that are trained on large amounts of text data.
Corpus	A corpus is a large and structured collection of textual data that is used to train and evaluate NLP models. The textual data in a corpus can come from a variety of sources, including books, newspapers, websites and other written or spoken materials.
Deep neural network	A deep neural network is a neural network with a

	certain level of complexity, specifically a neural network with more than two layers. Deep neural networks use sophisticated mathematical modelling to process data in complex ways. Deep neural networks are inspired by the structure and function of the human brain and are designed to learn and make predictions based on large amounts of input data.
Encoder architecture	An encoder architecture is a type of neural network architecture that is used to represent text data in a compact and meaningful way. The goal of an encoder architecture is to learn a dense, continuous representation of the input text that can be used as input to other NLP models, such as decoders or classifiers.
Features	Features refer to the characteristics of textual data that are used to represent it and make it appropriate for processing and analysis. These features can be either linguistic or statistical and are typically extracted from raw text data to facilitate tasks such as text classification.
Fine-tuning	Fine-tuning refers to the process of adapting a pre-trained language model to a specific task or domain by training the model on a smaller dataset that is related to the task or domain. The

	pre-trained model is usually a very large language model trained on a massive corpus of text.
Hyperparameters	A hyperparameter is a parameter that is set before training a model and cannot be learned from the training data. Hyperparameters play a critical role in determining the performance of a model and have a direct impact on its behaviour.
Labelling	Labelling refers to the process of annotating textual data with semantic information. This annotation process allows for the creation of annotated datasets that can be used to train and evaluate NLP models. Labelling is an important step in NLP as it provides the model with the ground truth information it needs to learn and make predictions.
Machine learning	Machine learning is a subfield of artificial intelligence that focuses on the development of algorithms and statistical models that enable computer systems to learn from data without being explicitly programmed.
Masked Language Modeling (MLM)	Masked Language Modeling is a technique used in NLP that involves masking or hiding certain words in a sentence and then training a model to predict what those words should be based on the

	context of the sentence.
Model	A model is a mathematical representation of a process or system that can be used to make predictions or perform tasks related to language. Models are trained on large amounts of labelled textual data and can be used to perform a variety of tasks such as text classification.
Next Sentence Prediction	Next Sentence Prediction is an NLP technique that involves training a model to predict whether two sentences are likely to follow each other in a given context. This task is typically framed as a binary classification problem, where the model must predict whether a given pair of sentences are either ‘related’ or ‘not related’ in terms of their meaning and context.
Prediction	A prediction refers to the output of a model that is produced in response to a given input. The input can be a sentence, a document, or any other textual data. The prediction is usually a label, a category, a probability distribution, or some other form of output that the model was trained to produce. Predictions in NLP are produced by applying the trained model to the input and computing the output based on the learned hyperparameters and the input features.

Token	A token is a sequence of characters that represent a single semantic unit in the input text. Tokens are the basic building blocks of NLP processing and are used to represent words, punctuation marks and other elements of the input text.
