# Supplementary material for: Automating the Identification of Feedback Quality Criteria and the CanMEDS Roles in Written Feedback Comments Using Natural Language Processing
Source: Perspect Med Educ. 2023 Dec 18;12(1):540–9. doi: 10.5334/pme.1056 (PMC10742245; doi:10.5334/pme.1056)
Supplement: Appendix B. — Structured categorisation matrix quality criteria. [file pme-12-1-1056-s2.pdf]

Appendix B. Structured categorisation matrix quality criteria

**Code**	**Definition**
Performance	The task on which the feedback is provided. This task must be observable.
Judgment	An adjective used to denote the gap between the performance and a standard. It is an evaluative statement expressed as a judgment on past performance.
Elaboration	Informative statement that builds further on the judgment expressed as a question, confirmation, suggestion, or justification… (e.g. it indicates what went well or did not go well, why it went well or not…). In doing so, it gives more details about the judgment.
Improvement	The aim of feedback is to improve the trainee's performance. The feedback must suggest strategies for improvement.
