# Supplementary material for: Automating the Identification of Feedback Quality Criteria and the CanMEDS Roles in Written Feedback Comments Using Natural Language Processing
Source: Perspect Med Educ. 2023 Dec 18;12(1):540–9. doi: 10.5334/pme.1056 (PMC10742245; doi:10.5334/pme.1056)
Supplement: Appendix C. — Cohen’s Kappa values for manual labelling. [file pme-12-1-1056-s3.pdf]

Appendix C. Cohen's Kappa values for manual labelling

Code	Cohen's Kappa value
Quality criteria	
Performance	0.77
Judgment	0.68
Elaboration	0.24
Improvement	0.83
CanMEDS roles	
Medical Expert	0.63
Communicator	0.62
Collaborator	0.60
Leader	0.51
Health Advocate	0.50
Scholar	0.63
Professional	0.21
