# Supplementary material for: Automating the Identification of Feedback Quality Criteria and the CanMEDS Roles in Written Feedback Comments Using Natural Language Processing
Source: Perspect Med Educ. 2023 Dec 18;12(1):540–9. doi: 10.5334/pme.1056 (PMC10742245; doi:10.5334/pme.1056)
Supplement: Appendix D. — Data distribution regarding the feedback quality criteria and CanMEDS roles throughout the training, development and test dataset. [file pme-12-1-1056-s4.pdf]

Appendix D. Data distribution regarding the feedback quality criteria and CanMEDS roles throughout the training, development and test dataset.

Label	# sentences in which the label occurred (%)			
**Feedback quality criteria**	**Training set**	**Development set**	**Test set**	**Total**
Performance	3,961 (45.45)	860 (46.04)	832 (44.54)	5,653 (45.40)
Judgment	3,251 (37.30)	709 (37.96)	688 (36.83)	4,648 (37.33)
Elaboration	622 (7.14)	146 (7.82)	129 (6.91)	897 (7.20)
Improvement	2,801 (32.14)	559 (29.93)	606 (32.44)	3,966 (31.85)
**CanMEDS roles**				
Medical Expert	4,084 (46.86)	837 (44.81)	856 (45.82)	5,777 (46.39)
Communicator	989 (11.35)	216 (11.56)	204 (10.92)	1,409 (11.32)
Collaborator	779 (8.94)	163 (8.73)	171 (9.15)	1,113 (8.94)
Leader	237 (2.72)	44 (2.36)	47 (2.52)	328 (2.63)
Health Advocate	247 (2.83)	56 (3.00)	66 (3.53)	369 (2.96)
Scholar	870 (9.98)	182 (9.74)	201 (10.76)	1,253 (10.06)

Professional	377 (4.33)	77 (4.12)	87 (4.66)	541 (4.34)
